# *ALK* in Non-Small Cell Lung Cancer (NSCLC) Pathobiology, Epidemiology, Detection from Tumor Tissue and Algorithm Diagnosis in a Daily Practice

**DOI:** 10.3390/cancers9080107

**Published:** 2017-08-12

**Authors:** Paul Hofman

**Affiliations:** 1 Laboratory of Clinical and Experimental Pathology, Pasteur Hospital, 30 avenue de la voie romaine, 06001 Nice CEDEX 01, France; hofman.p@chu-nice.fr; Tel.: +33-049-203-8855; Fax: +33-049-203-8850; 2 FHU OncoAge, Côte d’Azur University, 30 avenue de la voie romaine, 06001 Nice CEDEX 01, France; 3 Hospital-integrated Biobank (BB-0033-00025), 30 avenue de la voie romaine, 06001 Nice CEDEX 01, France

**Keywords:** *ALK* rearrangement, lung cancer, biology, immunohistochemistry, FISH, molecular biology

## Abstract

Patients with advanced-stage non-small cell lung carcinoma (NSCLC) harboring an *ALK* rearrangement, detected from a tissue sample, can benefit from targeted *ALK* inhibitor treatment. Several increasingly effective ALK inhibitors are now available for treatment of patients. However, despite an initial favorable response to treatment, in most cases relapse or progression occurs due to resistance mechanisms mainly caused by mutations in the tyrosine kinase domain of *ALK*. The detection of an *ALK* rearrangement is pivotal and can be done using different methods, which have variable sensitivity and specificity depending, in particular, on the quality and quantity of the patient’s sample. This review will first highlight briefly some information regarding the pathobiology of an *ALK* rearrangement and the epidemiology of patients harboring this genomic alteration. The different methods used to detect an *ALK* rearrangement as well as their advantages and disadvantages will then be examined and algorithms proposed for detection in daily routine practice.

## 1. Introduction

Among the genomic alterations present in non-small cell lung carcinoma (NSCLC) the *ALK* rearrangement is one that results in targeted therapy and in most cases gives a therapeutic response that prolongs the life of patients [1]. Thus, several molecular therapies targeting the *ALK* rearrangement have been developed or are being developed and are often effective, but obtain variable results for survival [2,3]. For example, crizotinib is a potent and selective ATP-competitive inhibitor of ALK tyrosine kinases. It received Food and Drug Administration (FDA) approval in the USA in 2011, and European Medicines Agency approval in 2012 [4]. However, despite its clinical efficacy, resistance to crizotinib invariably develops [5]. There is now a next generation of ALK inhibitors, including two that have been approved-ceritinib and alectinib-and others that are in development such as brigatinib and lorlatinib [3,6,7,8,9,10]. Moreover, ceritinib and the other next-generation ALK inhibitors are more potent than crizotinib and can overcome tumor cell resistance mechanisms [5]. Taken altogether, these results highlight the need to perform rapid and highly sensitive screening for an *ALK* rearrangement in NSCLC patients, so that new drugs can be appropriately administered.

An *ALK* rearrangement is detected in 3 to 7% of patients with stage IIIB/IV NSCLC, depending on the series and also probably according to the selection of the patients for molecular testing; in most cases it concerns an adenocarcinoma [11,12,13,14,15]. The frequency of this genomic alteration is higher if only non-smoker patients are considered, from 17 to 20%, depending on the series [11,12]. Relapse or tumor progression is systematically noted at a more or less short term, which may lead to a change in the targeted therapy [16,17]. The major cause of this change results from the emergence of mutations in the *ALK* gene though other mechanisms are possible [18,19,20].

In view of the therapeutic consequences of the detection of an *ALK* rearrangement several methodological approaches using tissue or cell samples have progressively been developed. Fluorescence in situ hybridization (FISH) was the first to be described. Immunochemistry and molecular biology approaches such as reverse transcriptase-polymerase chain reaction (RT-PCR) or new generation sequencing (NGS) were then developed. The use in clinical practice of one or several of these methods for a single patient raises a number of questions, in particular the sensitivity and specificity of the method. In fact, the reduced size of tissue samples obtained for morphological diagnosis of NSCLC and the increase in the number of cytological samples (associated or not with tissue biopsies) has led to new strategies for optimal handling of biological material and to methods of detection with these samples. The emergence of *ALK* mutations during treatment also raises the question of access to new tissue and/or cell samples for its detection, in particular by methods in molecular biology.

After briefly covering the biology of lung cancers associated with an *ALK* rearrangement, the consequences of this rearrangement in cells, the epidemiology of lung cancer, this review will examine the different analytical methods that detect this genomic alteration, as well as their advantages and limits, and will present algorithms for diagnosis in daily practice.

## 2. The *ALK* Rearrangement in Lung Cancer: Mechanism and Consequences

The *ALK* rearrangement leads to constitutive expression of an oncogenic fusion protein, initially detected in NSCLC [21,22,23]. At the cellular level, ALK regulates canonical signaling pathways that are shared with other receptor tyrosine kinases including RAS-mitogen-activated protein kinase, phosphoinositide 3-kinase-AKT, and JAK-STAT pathways. When there are some *ALK* rearrangements, 5′ end partners such as EML4 and NPM are fused to the intracellular tyrosine kinase domain of ALK. The consequence is aberrant expression of the ALK fusion protein in the cytoplasm. The different domains in the partner proteins promote dimerization and oligomerization of the fusion proteins, inducing constitutive activation of the ALK kinase and its downstream signaling pathways [21,22,23]. The consequence is uncontrolled cellular proliferation and survival. More than twenty *ALK* fusion partners have been described [24]. The breakpoints on the *ALK* gene almost always occur in intron 19 and, rarely, in exon 20, resulting in a constant inclusion of the *ALK* kinase domain in the fusion gene. A common feature of the fused partner genes is the presence of a basic coil-coil domain, which allows the dimerization of the fusion proteins. Moreover, *EML4-ALK*, the most common *ALK* fusion found in NSCLC, is formed by an inversion occurring on the short arm of chromosome 2 involving the genes encoding *ALK* (2p23) and *EML4* (2p21) with variants 1, 2, and 3a/3b being the most frequent fusion patterns [25,26]. The three major variants (v1: E13; A20, v2: E20; A20, and v3; E6; A20) account for more than 90% of lung cancers associated with *EML4-ALK*. In *ALK* translocated NSCLC, *EML4* does not appear to be the exclusive fusion partner with *ALK*. Two other fusions have been described, *TFG* and *KIF5B* [27,28]. Both were identified as an *ALK*-fusion partner from NSCLC tumor samples and the two proteins also fuse with the intracellular domain of ALK [27,28]. It is noteworthy that the presence of these non-*EML4* fusion partners for *ALK* can have implications for the method used for detection of *ALK* translocated NSCLC in daily practice. Because the gene rearrangement involves a large chromosomal inversion and translocation, FISH was the first method used for detection of *ALK* rearrangement. Mutations in the *ALK* gene result in decreased binding of an inhibitor, such as crizotinib, or increased ATP binding affinity [29]. Moreover, other resistance mechanisms have been identified such as the activation of EGFR and KRAS pathways by their respective mutations, *ALK* and *KIT* gene amplification, and more recently *YES1* mutations [29,30].

## 3. Epidemiology of Lung Cancers with an *ALK* Rearrangement

The percentage of patients with an *ALK* rearrangement varies from 1% to 5% depending on the population, and if all the histological types of NSCLC are taken into consideration or not for evaluation of the presence of an *ALK* rearrangement, while disregarding the smoking status [11,31,32,33,34]. In general, this genomic alteration if more often detected in young and non or past smokers [35,36]. So the frequency of an *ALK* rearrangement can be higher than 17% for non-smoker and young patients [11,37]. It is a biomarker of poor prognosis in a population of non-smoker patients [38]. This genomic alteration is essentially detected in adenocarcinomas and only very occasionally in epidermoid carcinomas or other rare histological types of NSCLC [11,39,40,41]. *ALK* rearrangement is more frequent in acinus forms of adenocarcinomas of Asian patients and in signet-ring cell adenocarcinomas of Caucasian patients [28,37]. Most *ALK*-positive lung adenocarcinomas show areas of solid tissue with 10% signet-ring cells [42]. The fact that an *ALK* rearrangement can exist in certain patients with an epidermoid carcinoma raises the question of systematically analyzing for this modification in this histological type [43]. The presence of an *ALK* rearrangement almost always excludes the presence of other genomic alterations associated in particular with mutations in *KRAS* and *EGFR* [44]. However, exceptional cases associated with other mutations have been reported [39]. It is noteworthy that some studies reported *ALK* translocation in non-tumor tissue, but below the accepted thresholds for determined the *ALK* status positivity [45].

## 4. Methods for Detection of an *ALK* Rearrangement

Different methods can be used to define the *ALK* status in tumor tissues. The most frequently employed methods include immunohistochemistry (IHC) and immunocytochemistry (ICC), FISH, real-time polymerase chain reaction (RT-PCR) and NGS. A number of studies, described in detail below, have shown discordant results when comparing these approaches, which can limit systematic use [45,46,47,48]. The FISH reasons for this are numerous. The first concerns the sample itself and the variable pre-analytical handling of the sample. Thus, bad fixation (delay in fixation, hyper or hypofixation, inappropriate fixative solution) can have a substantial and variable impact on the detection level of the expression of the ALK protein and/or the quality of the ALK RNA. One other reason lies in the heterogeneity of the expression of this genomic alteration within the tissue [49]. However, the most frequent cause of discrepancies between the different techniques concerns the variable sensitivity of these approaches, notably linked for the quality and quantity of the RNA obtained after extraction.

### 4.1. Immunohistochemistry and Immunocytochemistry Methods

IHC is a method used in all pathology laboratories and thus highly accessible [50]. It is possible to rapidly evaluating the *ALK* status with IHC on formalin fixed tissue sections, which is an easy approach that does not require a lot of technical or medical expertise. Several antibodies for the detection of the expression of ALK in tissues have been commercialized, in particular the 54A (Novocastra, Leica Biosystems, Buffalo Grove, IL, USA) and D5F3 (Cell Signaling Technology, Danvers, MA, USA) clones, which are the most used and reliable [51,52,53,54]. Certain antibodies, such as ALK1 (Dako) are not recommended and others like 1A4 (Origene, Rockville, MD, USA) must be used with precaution due to their variable specificity [55,56]. Overall, the D5F3 clone seems to give the most satisfactory results and is now approved by the FDA as a companion diagnostic test for ALK inhibitors treatment. Thus, patients with a positive ALK IHC can be treated with any specific ALK inhibitor. Several studies have also validated the use of anti-ALK antibodies with cytological material. ICC has essentially been developed with formalin fixed samples embedded in paraffin, as cellblocks [57,58,59,60].

Thus, IHC with anti-ALK holds several advantages. Aside from those described above, it is accepted that even after a too long fixation time, the stability of the protein allows detection of most cases harboring an *ALK* rearrangement.

To obtain a reliable interpretation certain pitfalls must be avoided such as false positive results close to necrotic zones, in the event of tyramide signal amplification and if positive alveoli macrophages are mistaken for tumor cells. Heterogeneous labeling can also be seen with certain surgical resected specimens, in particular in the center of large tumors, subsequent to a delay in fixation. A delay in fixation also has an impact on the interpretation of ALK IHC when performed on tissue microarrays with cores removed at random from the center of a tumor that has had a delay in fixation with formalin. Thus, within the context of a quality process and accreditation of an IHC test, the validation of the method and inter-laboratory controls, which includes external evaluation of the quality, are important for this predictive marker of therapeutic response [61].

### 4.2. The FISH Method

FISH was the first method used to detect an *ALK* rearrangement, foremost in tissue biopsies and then on cytological material. Initially it was considered to be the only gold standard method. Several probes can be used to visualize this rearrangement. Regardless of the probe, the cutoff for a positive result is 50 tumor cells with the rearrangement. FISH was the most frequently used method until the commercialization of anti-ALK antibodies and most laboratories continue to use this approach, either as a first-line test or to validate a positive IHC or ICC result [62,63]. However, compared to IHC this method has a number of disadvantages. It requires more time to perform and necessitates special equipment in addition to substantial technical and analytical expertise [64]. The observed signal is sometimes difficult to interpret or ambiguous and, in principle, more than a hundred tumor cells are required to obtain a reliable result, which may not be the case for small-sized tissue or cytological samples [65]. Not all pathology laboratories have a fluorescence microscope or the equipment for the hybridization steps. Additionally, rare false negative and false positive results can occur with FISH for detection of *ALK* rearrangements. In this case, certain rearrangements give rare and particular patterns [66]. In the event of an ambiguous FISH analysis, some complementary probes can be used [67]. False negative results can arise from a small number of tumor cells in the sample for analysis (in particular those with less than 50 tumor cells), from reactive epithelial cells or normal or abnormal cells mistaken for cancer cells or from difficult to interpret signals resulting from formalin hyper- or hypo-fixation. False positive results arising from atypical patterns due to multiple fusion of signals or a single green signal with 5′ centromeric probes.

### 4.3. RT-PCR Method

An approach using RT-PCR for detection of an *ALK* rearrangement in tissue and cell samples has been proposed [68,69]. The sensitivity and specificity varies depending on the different variables of the detection panel used but also on the quantity and quality of the extracted RNA. This method requires technical and medical expertise to obtain and interpret the results. Moreover, to eliminate sampling error and potential false negative results, visual control of the morphology is required to ensure the presence and percentage of tumor cells in the tissue or cytological sample. A multiplex RT-PCR-based assay can detect with sensitivity certain *ALK* fusion gene variants, although reproducible RT-PCR results are difficult to obtain with FFPE tissue sections [70,71]. RT-PCR is a sensitive approach for detection of some *EML4-ALK* variants but not all. In particular, this technique allows the detection of the *EML4-ALK* variant 1, 2 and 3a/b. One advantage of RT-PCR, in contrast to IHC and FISH, is that this technique is free from subjectivity in assessment of the analysis. Moreover, identification of the specific variant can be determined using RT-PCR, which could be important in predicting patient response to ALK inhibitors. In this context, different additional primer sets can be added to the RT-PCR methodology to detect some rare reported variants. Therefore RT-PCR analysis must definitively be multiplexed. For this there are at least 13 variants *EML4-ALK* fusions and some non-*EML4* translocation partners [26,68]. In this context, any PCR based strategy must incorporate validated primer pairs for all known *ALK* fusions to avoid false negative results. Moreover, more than 20 putative fusion partners of ALK have been described so fare. Therefore, the diagnosis can be certainly difficult to interpret in some cases when using PCR methods. A few publications show that RT-PCR based detection of *EML4-ALK* can yield positive results in the absence of detectable *ALK*-rearrangements in both tumor and non-tumor tissues [45]. Finally, the clinical utility of RT-PCR should always be evaluated with regard to the treatment response in clinical studies.

### 4.4. NGS Approach

Analysis by NGS in molecular pathology laboratories has revolutionized the care of advanced-stage NSCLC. A single analysis is capable of detecting a substantial number of mutations on variable panels of genes [72,73]. Thus, due to the potential of the NGS technique it is now present in most molecular pathology laboratories but certain hurdles continue to be present and discussed [74,75,76,77]. It is indeed important to consider not only the quantity and the percentage of tumor cells used for this type of analysis, but also the total amount of tissue and the total number of cells present in the sample. Depending on the NGS technique used, the minimal amount of nucleic acid varies from 5 to 10 ng (around 1000 tumor cells) for the NGS technique based on PCR, and, 100 to 200 ng for the NGS technique based on hybrid capture. In fact, since this latter technique requires at least 100 ng of RNA of good quality more than a third of the samples cannot be used to give reliable results [76]. The detection of an *ALK* rearrangement can be associated with examination for fusions on the *ROS1* and *NTRK* genes, which avoids sequential analyses that are costly, time consuming and require additional biological material [47,78]. Several recent studies describe the potential of the NGS technique for the detection of an *ALK* rearrangement in routine practice, but a number of [79,80]. As an example, Qiagen (Hilden, Germany) recently launched the GeneReader NGS system offering soon a complete solution (called the GeneRead™ QIAact Lung All-in-One Assay) for both detection of mutation and different rearrangements. In particular, a targeted panel will cover the fusion genes of interest for NSCLC (including *ALK*, *ROS1*, *NTRK1*, *RET*) will be launched at the end of 2017 [81]. However a number of pitfalls and/or limitations of NGS approach for *ALK* rearrangement detection need to be pointed out. The vast majority of lung cancer biopsy specimens from patients are stored in formalin fixed paraffin embedded (FPPE) tissues. As for RT-PCR, the quality of the RNA extracted from material fixed in formalin needs to be considered since too much degradation can lead to false negative results. As seen above, NGS can also require a certain amount of extracted RNA depending on the approach and the panel of analyses. These techniques require strong technical and medical expertise and in general need more time than other methods, thus leading to variable delays in obtaining a response, which depends on the laboratory and the method. In fact, we have to thinks that presently it is recommended that an *ALK* result be provided for the physicians within 10 days after the performed biopsy [82]. An additional advantage of NGS is the detection of potential presence of some *ALK* mutations, in particular when considering survival of patients on ALK inhibitors. These mutations are the main reason for progression or relapse of patients on specific ALK inhibitors. It is of interest to note that depending on the inhibitor used certain mutation in the *ALK* gene emerge more frequently and resistance to a specific inhibitor requires a quick change in inhibitor [18].

### 4.5. Other Methods

A multiplexed transcript-based assay (nCounter, nanoString, Seattle, WA, USA) has been developed for simultaneous detection of multiple gene fusions. In particular, this approach has been developed for detection of an *ALK* rearrangement [83]. Briefly, this assay is based on the dual hybridization of a capture and a molecularly bar-codes reporter probe complementary to a contiguous target sequence [84]. Moreover, a single-tube test for *ALK, ROS1* and *RET* fusions has been recently developed [85]. This approach seems to have the potential for a cost-effective assay in daily practice. Moreover, the nanoString nCounter can detect targetable gene fusions on a variety of specimens from surgical resection to small biopsies or cytology cell blocks. Globally, around 20% tumor cellularity is required for detection, but the detection of an *ALK* fusion transcript could be possible in samples with as low as 5% tumor cellularity. Moreover, the fixation method does not influence the performance of the nanoString approach. Finally, nanoString might provide additional information complementary to IHC and FISH. In situ hybridization with RNA has also been described for evaluation of the tissue expression of an *ALK* rearrangement [86,87,88]. In fact, the RNA ISH method (RNAscope) is a rapid technique, with quite easy handling, similar to IHC. It can semi quantitatively assess the mRNA signal in target cells with conventional light microscopy [88]. Finally, multiplex IHC analyses are also being developed to analyze in parallel the *ALK* status and the expression of certain checkpoint inhibitors, and these analyses can participate in studies into novel therapeutic strategies [89].

### 4.6. Comparatives Analyses

Taken the number of different approaches available for the evaluation of the *ALK* status in the daily practice, several comparative studies have evaluated their sensitivity and specificity. Other considerations such as the costs, the procedural difficulties and the resolution should be also part of these comparisons (Table 1). Several studies have compared IHC, in particular using the D5F3 clone, and *ALK* FISH, which show very good concordant results with a sensitivity of 81 to 100% and a specificity of 82 to 100%, depending on the study [62,90,91,92,93,94]. However, these results must take into consideration the intensity of the label and only the strong label (3+) concords at 100% with the *ALK* results obtained with FISH [95]. Thus, discordant results have been also reported for these different methods [46,48,96,97,98,99,100]. These discordant results were ALK-positive with IHC and *ALK*-negative with FISH. This can be explained by false negative FISH results from difficult to interpret samples and a positive result from less than 20% tumor cells [65,99]. FISH does not detect all *ALK* rearrangements and certain complex rearrangements reduce the distance between the two FISH probes, which can give a false negative FISH image [46]. Aside from the difference in sensitivity and specificity the discord can sometimes be explained by the intra- and inter-tumor heterogeneity in terms of the *ALK* status, in particular when comparing IHC/ICC or FISH and methods using direct extraction of RNA without prior visual control of the lesion [49,101,102]. Certain isoforms of *ALK* arise from a mechanism of alternative initiation of transcription giving protein expression that is not *ALK*-positive with FISH [103]. It is important to mention that some patients ALK-positive with IHC and *ALK*-negative with FISH can respond to treatment with crizotinib [104,105]. A negative IHC result can be observed in cases of *ALK* amplification [106,107]. In the same way, negative FISH results can be associated with positive RT-PCR results [108]. Some studies have compared the specificity and sensitivity of IHC, FISH, RT-PCR and/or NGS [109,110,111]. Finally, other studies have shown very good concordance of the results obtained with the nanoString testing with IHC and FISH, while some discrepancy was observed in other studies [112,113,114].

In conclusion, although correlation between the results for ALK IHC and *ALK* FISH is excellent, as a predictive marker for response to ALK inhibitor therapy, IHC alone has recently been validated with the D5F3 IHC assay [115]. The RT-PCR method for fusion genes is a high-throughput screening tool with quite a rapid turnaround time. However, this method is not able to identify rearrangements involving unknown fusion partners. NGS probably represents a more practical and reliable *ALK* testing approach for use in clinical routine practice. Moreover, this method can assess genomic-related mechanisms of resistance to *ALK*-targeted therapies.

## 5. Algorithms for Diagnosis of an *ALK* Rearrangement

**Figure 1:**
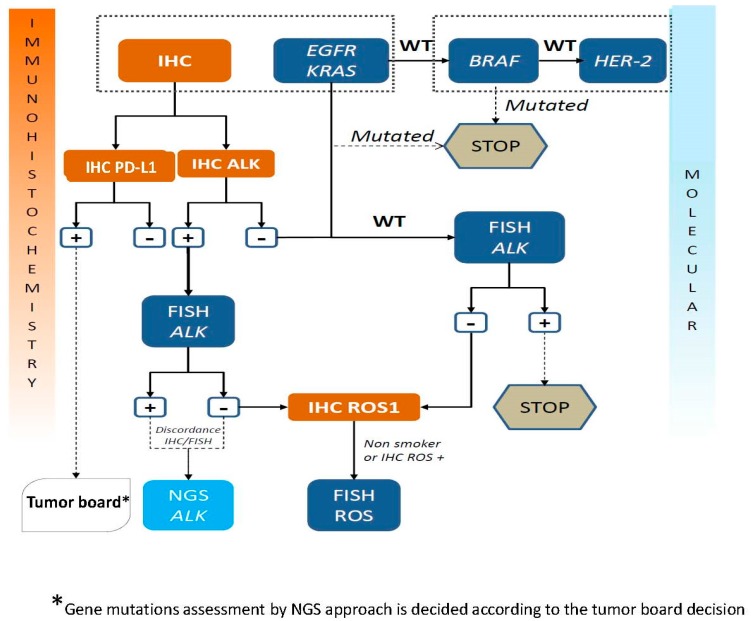
Current algorithm proposed at the LPCE (Nice Hospital, France) for *ALK* testing [incorporating into the standard of care *ROS1* status and *Epidermal Growth Factor Receptor* gene (*EGFR)* mutation testing in lung adenocarcinoma].

The substantial number of technological approaches for detection of an *ALK* rearrangement raises the question of their complementarity or lack of complementarity and the necessity to see development of different approaches in the same laboratory so as to reply to the needs of physicians [116,117]. It is possible to question if these approaches can be used in a sequential fashion or in combination. In fact, the samples from NSCLC patients sent to the pathology laboratories are smaller and smaller and thus require strategies that economize the amount of biological material for the morphological, IHC and molecular biology analyses [118]. Moreover, it is important to rapidly transfer reliable results, which requires sensitive and specific tests [119]. Therefore, several algorithms have been proposed [53,104,120,121,122,123,124,125]. At present the extensively employed algorithm consists in first-line ALK IHC (or ICC) and if the result is positive FISH analysis is used for confirmation. A negative IHC does not lead to other analyses and the *ALK* status is considered to be negative. A second algorithm with first-line ALK IHC/ICC and then if positive NGS analysis can be proposed for confirmation [109,110]. Algorithm may also depend of the country, the local organization and the resources (personal, platform) available in each institution. One possible algorithm is shown in Figure 1. In this context, it is necessary to mention that companion diagnostic tests (CDx) are gatekeepers with respect to the treatment decision for patient with life-threating conditions [126]. Physicians must always require high standards for introduction of new analytical methods and technologies. Therefore, it is noteworthy to mention that so far, in USA, the ALK (D5F3) CDx IHC assay and the FISH *ALK* break apart assay are the only assays that have obtained regulatory approval. In this context, if other assays, such as those based on RT-PCR, NGS and the Nanostring approach, should be used as CDx for ALK inhibitor treatments, it will be mandatory to do extensive analytical and clinical validation studies as well as some ring studies with external quality assessment. However, CDx, which obtained regulatory approval, is not always perfect. As an example, for FISH analysis, the FDA recommended counting at least 50 tumor cells for *ALK* status assessment, and if 15–25 cells demonstrated an *ALK* rearrangement, an additional 50 tumor cells have to be counted by another pathologist. However, it is quite obvious that it is not an ideal approach and some pitfalls and/or errors may occur for different reasons [127].

## 6. Conclusions

To provide patients with advanced-stage NSCLC with ALK inhibitors it is essential to systematically analyze for *ALK* rearrangements using a rapid, cost effective and reliable approach [1,3]. For a long time the FISH technique was the only method for evaluation of the *ALK* status. However, FISH analysis is now considered to be labor-intensive, quite expensive and difficult to implement systematically in all pathology laboratories as a screening and diagnostic assay. Moreover, the discordant results comparing IHC and FISH are problematic and are quite frequent in cases with borderline FISH positivity (15–20% split nuclei). At present, IHC/ICC is the first to be used, in particular when the sample is small and/or contains few cells [128]. Moreover, it has been suggested that ALK IHC is better than *ALK* FISH at predicting response to ALK inhibitors [123]. However, occasionally there is FISH-IHC discordance that may make difficult the determination of the *ALK* status. Confirmation of the IHC/ICC ALK result must be made by another method. The NGS approach, except for the *ALK* rearrangement, can detect other rearrangements including with *ROS1, RET* and *NTRK*, all in a single analysis, which avoids sequential investigation of these genomic alterations and thereby gains precious time for administration of effective treatment. However, depending on the amount of tumor cells and the quality of the nucleic acid, these techniques need to be discussed on a case-by-case basis, before arriving at a negative result. Finally, it is very important, disregarding the method used, to assure the quality of the results. Therefore, participation in external quality control and inter-laboratory control and ring studies is indispensable. This should lead to accreditation tests based on the norms of the country in which these theranostic tests are performed [61,129,130,131,132].

## Figures and Tables

**Table 1:** Comparison of different methods for *ALK* testing in tissue sample.

Parameters	FISH	IHC	qRT-PCR	NGS	NanoString
**Sample criteria**					
**RNA input required**	**NA**	**NA**	**+**	**+++**	**++**
**RNA quality**	**++**	**+++**	**+**	**+**	**++**
**% of tumor cells**	**+**	**+++**	**++**	**+**	**+**
**Sensitivity**	**+++**	**+++**	**++**	**++**	**++**
**Specificity**	**+++**	**+++**	**++**	**+++**	**++**
**Costs**					
**Reagents**	**++**	**+++**	** ++**	** +**	** +++**
**Device/hardware/software**	**++**	**+++**	**++**	**+**	**+**
**Resolution**					
**Quantitative precision**	**+**	**+**	**++**	**+++**	**+++**
**Accuracy at low concentrations**	**NA**	**NA**	**++**	**+**	**+++**
**Variants detection**	**NA**	**NA**	**++**	**+++**	**++**
**TAT**					
**Hands on time**	**+**	**+++**	**++**	**+**	**+++**
**Results analysis**	**+**	**+++**	**++**	**+**	**++**
**Interpretation**	**+**	**+++**	**++**	**++**	**++**
**Throughput**	**+**	**++**	**++**	**+++**	**+++**

TAT: turnaround time; NA: not applicable; +: worse approach; ++: intermediate option; +++: best approach.

## References

[1] Shaw A.T., Engelman J.A. (2013). ALK in lung cancer: Past, present, and future. J. Clin. Oncol..

[2] Ilie M., Hofman P. (2016). Expanding opportunities for crizotinib resistance in ALK-positive lung cancer patients. Transl. Cancer Res..

[3] Peters S., Camidge D.R., Shaw A.T., Gadgeel S., Ahn J.S., Kim D.W., Ou S.I., Pérol M., Dziadziuszko R., Rosell R. (2017). Alectinib versus crizotinib in untreated ALK-positive non-small-cell lung cancer. N. Engl. J. Med..

[4] Di Maio M., de Marinis F., Hirsch F.R., Gridelli C. (2014). Diagnostic and therapeutic issues for patients with advanced non-small cell lung cancer harboring anaplastic lymphoma kinase rearrangement: European vs. US perspective (review). Int. J. Oncol..

[5] Qian M., Zhu B., Wang X., Liebman M. (2017). Drug resistance in ALK-positive non-small cell lung cancer patients. Semin. Cell Dev. Biol..

[6] Khozin S., Blumenthal G.M., Zhang L., Tang S., Brower M., Fox E., Helms W., Leong R., Song P., Pan Y. (2015). FDA approval: Ceritinib for the treatment of metastatic anaplastic lymphoma kinase-positive non-small cell lung cancer. Clin. Cancer Res..

[7] Kim D.W., Tiseo M., Ahn M.J., Reckamp K.L., Hansen K.H., Kim S.W., Huber R.M., West H.L., Groen H.J.M., Hochmair M.J. (2017). Brigatinib in patients with crizotinib-refractory anaplastic lymphoma kinase-positive non-small-cell lung cancer: A randomized, multicenter phase II trial. J. Clin. Oncol..

[8] Larkins E., Blumenthal G.M., Chen H., He K., Agarwal R., Gieser G., Stephens O., Zahalka E., Ringgold K., Helms W. (2016). FDA approval: Alectinib for the treatment of metastatic, ALK-positive non-small cell lung cancer following crizotinib. Clin. Cancer Res..

[9] Markham A. (2017). Brigatinib: First global approval. Drugs.

[10] Santarpia M., Daffinà M.G., D’Aveni A., Marabello G., Liguori A., Giovannetti E., Karachaliou N., Gonzalez Cao M., Rosell R., Altavilla G. (2017). Spotlight on ceritinib in the treatment of ALK+ NSCLC: Design, development and place in therapy. Drug Des. Devel. Ther..

[11] Barlesi F., Mazieres J., Merlio J.P., Debieuvre D., Mosser J., Lena H., Ouafik L., Besse B., Rouquette I., Westeel V. (2016). Routine molecular profiling of patients with advanced non-small-cell lung cancer: Results of a 1-year nationwide programme of the French Cooperative Thoracic Intergroup (IFCT). Lancet.

[12] Le T., Gerber D.E. (2017). ALK alterations and inhibition in lung cancer. Semin. Cancer Biol..

[13] Schildgen V., Lochi V., Lüsebrink J., Brockmann M., Schidgen O. (2012). Epidemiology of EML4-ALK translocations in a small German NSCLC patient cohort. Per. Med..

[14] Schildgen V., Lüsebrink J., Schildgen O., Stoelben E., Brockmann M. (2016). Epidemiology of KRAS, BRAF, and EGFR mutations in lung cancer in a German tertiary hospital in patients with testing indication. Per. Med..

[15] Schildgen V., Schildgen O. (2016). Routine molecular profiling of patients with NSCLC. Lancet.

[16] Dagogo-Jack I., Shaw A.T. (2016). Crizotinib resistance: Implications for therapeutic strategies. Ann. Oncol..

[17] Lin J.J., Riely G.J., Shaw A.T. (2017). Targeting ALK: Precision medicine takes on drug resistance. Cancer Discov..

[18] Gainor J.F., Dardaei L., Yoda S., Friboulet L., Leshchiner I., Katayama R., Dagogo-Jack I., Gadgeel S., Schultz K., Singh M. (2016). Molecular mechanisms of resistance to first- and second-generation ALK Inhibitors in ALK-rearranged lung cancer. Cancer Discov..

[19] Isozaki H., Ichihara E., Takigawa N., Ohashi K., Ochi N., Yasugi M., Ninomiya T., Yamane H., Hotta K., Sakai K. (2016). Non-small cell lung cancer cells acquire resistance to the ALK inhibitor alectinib by activating alternative receptor tyrosine kinases. Cancer Res..

[20] Ou S.I., Lee T.K., Young L., Fernandez-Rocha M.Y., Pavlick D., Schrock A.B., Zhu V.W., Milliken J., Ali S.M., Gitlitz B.J. (2017). Dual occurrence of ALK G1202R solvent front mutation and small cell lung cancer transformation as resistance mechanisms to second generation ALK inhibitors without prior exposure to crizotinib. Pitfall of solely relying on liquid re-biopsy?. Lung Cancer.

[21] Allouche M. (2007). ALK is a novel dependence receptor: Potential implications in development and cancer. Cell Cycle.

[22] McDermott U., Iafrate A.J., Gray N.S., Shioda T., Classon M., Maheswaran S., Zhou W., Choi H.G., Smith S.L., Dowell L. (2008). Genomic alterations of anaplastic lymphoma kinase may sensitize tumors to anaplastic lymphoma kinase inhibitors. Cancer Res..

[23] Soda M., Choi Y.L., Enomoto M., Takada S., Yamashita Y., Ishikawa S., Fujiwara S., Watanabe H., Kurashina K., Hatanaka H. (2007). Identification of the transforming EML4-ALK fusion gene in non-small-cell lung cancer. Nature.

[24] Sasaki T., Rodig S.J., Chirieac L.R., Jänne P.A. (2010). The biology and treatment of EML4-ALK non-small cell lung cancer. Eur. J. Cancer.

[25] Woo C.G., Seo S., Kim S.W., Jang S.J., Park K.S., Song J.Y., Lee B., Richards M.W., Bayliss R., Lee D.H. (2017). Differential protein stability and clinical responses of EML4-ALK fusion variants to various ALK inhibitors in advanced ALK-rearranged non-small cell lung cancer. Ann. Oncol..

[26] Yoshida T., Oya Y., Tanaka K., Shimizu J., Horio Y., Kuroda H., Sakao Y., Hida T., Yatabe Y. (2016). Differential crizotinib response duration among ALK fusion variants in ALK-positive non-small-cell lung cancer. J. Clin. Oncol..

[27] Rikova K., Guo A., Zeng Q., Possemato A., Yu J., Haack H., Nardone J., Lee K., Reeves C., Li Y. (2007). Global survey of phosphotyrosine signaling identifies oncogenic kinases in lung cancer. Cell.

[28] Takeuchi K., Choi Y.L., Togashi Y., Soda M., Hatano S., Inamura K., Takada S., Ueno T., Yamashita Y., Satoh Y. (2009). KIF5B-ALK, a novel fusion oncokinase identified by an immunohistochemistry-based diagnostic system for ALK-positive lung cancer. Clin. Cancer Res..

[29] Lovly C.M., Pao W. (2012). Escaping ALK inhibition: Mechanisms of and strategies to overcome resistance. Sci. Transl. Med..

[30] Van der Wekken A.J., Saber A., Hiltermann T.J., Kok K., van den Berg A., Groen H.J. (2016). Resistance mechanisms after tyrosine kinase inhibitors afatinib and crizotinib in non-small cell lung cancer, a review of the literature. Crit. Rev. Oncol. Hematol..

[31] Ha S.Y., Choi S.J., Cho J.H., Choi H.J., Lee J., Jung K., Irwin D., Liu X., Lira M.E., Mao M. (2015). Lung cancer in never-smoker Asian females is driven by oncogenic mutations, most often involving EGFR. Oncotarget.

[32] Hong S., Fang W., Hu Z., Zhou T., Yan Y., Qin T., Tang Y., Ma Y., Zhao Y., Xue C. (2014). A large-scale cross-sectional study of ALK rearrangements and EGFR mutations in non-small-cell lung cancer in Chinese Han population. Sci. Rep..

[33] Lee B., Lee T., Lee S.H., Choi Y.L., Han J. (2016). Clinicopathologic characteristics of EGFR, KRAS, and ALK alterations in 6,595 lung cancers. Oncotarget.

[34] Vidal J., Clavé S., de Muga S., González I., Pijuan L., Gimeno J., Remón J., Reguart N., Viñolas N., Gironés R. (2014). Assessment of ALK status by FISH on 1000 Spanish non-small cell lung cancer patients. J. Thorac. Oncol..

[35] Rodig S.J., Mino-Kenudson M., Dacic S., Yeap B.Y., Shaw A., Barletta J.A., Stubbs H., Law K., Lindeman N., Mark E. (2009). Unique clinicopathologic features characterize ALK-rearranged lung adenocarcinoma in the western population. Clin. Cancer Res..

[36] Sacher A.G., Dahlberg S.E., Heng J., Mach S., Jänne P.A., Oxnard G.R. (2016). Association between younger age and targetable genomic alterations and prognosis in non-small-cell lung cancer. JAMA Oncol..

[37] Shaw A.T., Yeap B.Y., Mino-Kenudson M., Digumarthy S.R., Costa D.B., Heist R.S., Solomon B., Stubbs H., Admane S., McDermott U. (2009). Clinical features and outcome of patients with non-small-cell lung cancer who harbor EML4-ALK. J. Clin. Oncol..

[38] Yang P., Kulig K., Boland J.M., Erickson-Johnson M.R., Oliveira A.M., Wampfler J., Jatoi A., Deschamps C., Marks R., Fortner C. (2012). Worse disease-free survival in never-smokers with ALK+ lung adenocarcinoma. J. Thorac. Oncol..

[39] Alrifai D., Popat S., Ahmed M., Gonzalez D., Nicholson A.G., Parcq Jd., Benepal T. (2013). A rare case of squamous cell carcinoma of the lung harbouring ALK and BRAF activating mutations. Lung Cancer.

[40] Bolzacchini E., Tuzi A., Pinotti G. (2017). ALK-rearranged squamous cell carcinoma of the lung treated with two lines of ALK inhibitors. J. Thorac. Oncol..

[41] Zhao W., Choi Y.L., Song J.Y., Zhu Y., Xu Q., Zhang F., Jiang L., Cheng J., Zheng G., Mao M. (2016). ALK, ROS1 and RET rearrangements in lung squamous cell carcinoma are very rare. Lung Cancer.

[42] Popat S., Gonzalez D., Min T., Swansbury J., Dainton M., Croud J.G., Rice A.J., Nicholson A.G. (2012). ALK translocation is associated with ALK immunoreactivity and extensive signet-ring morphology in primary lung adenocarcinoma. Lung Cancer.

[43] Ochi N., Yamane H., Yamagishi T., Takigawa N., Monobe Y. (2013). Can we eliminate squamous cell carcinoma of the lung from testing of EML4-ALK fusion gene?. Lung Cancer.

[44] Gainor J.F., Varghese A.M., Ou S.H., Kabraji S., Awad M.M., Katayama R., Pawlak A., Mino-Kenudson M., Yeap B.Y., Riely G.J. (2013). ALK rearrangements are mutually exclusive with mutations in EGFR or KRAS: An analysis of 1683 patients with non-small cell lung cancer. Clin. Cancer Res..

[45] Martelli M.P., Sozzi G., Hernandez L., Pettirossi V., Navarro A., Conte D., Gasparini P., Perrone F., Modena P., Pastorino U. (2009). EML4-ALK rearrangement in non-small cell lung cancer and non-tumor lung tissues. Am. J. Pathol..

[46] Ali S.M., Hensing T., Schrock A.B., Allen J., Sanford E., Gowen K., Kulkarni A., He J., Suh J.H., Lipson D. (2016). Comprehensive genomic profiling identifies a subset of crizotinib-responsive ALK-rearranged non-small cell lung cancer not detected by fluorescence in situ hybridization. Oncologist.

[47] Abel H.J., Al-Kateb H., Cottrell C.E., Bredemeyer A.J., Pritchard C.C., Grossmann A.H., Wallander M.L., Pfeifer J.D., Lockwood C.M., Duncavage E.J. (2014). Detection of gene rearrangements in targeted clinical next-generation sequencing. J. Mol. Diagn..

[48] Ilie M.I., Bence C., Hofman V., Long-Mira E., Butori C., Bouhlel L., Lalvée S., Mouroux J., Poudenx M., Otto J. (2015). Discrepancies between FISH and immunohistochemistry for assessment of the ALK status are associated with ALK ‘borderline’-positive rearrangements or a high copy number: A potential major issue for anti-ALK therapeutic strategies. Ann. Oncol..

[49] Abe H., Kawahara A., Azuma K., Taira T., Takase Y., Fukumitsu C., Murata K., Yamaguchi T., Akiba J., Ishii H. (2015). Heterogeneity of anaplastic lymphoma kinase gene rearrangement in non-small-cell lung carcinomas: A comparative study between small biopsy and excision samples. J. Thorac. Oncol..

[50] Kim S.W., Roh J., Park C.S. (2016). Immunohistochemistry for Pathologists: Protocols, Pitfalls, and Tips. J. Pathol. Transl. Med..

[51] Conklin C.M., Craddock K.J., Have C., Laskin J., Couture C., Ionescu D.N. (2013). Immunohistochemistry is a reliable screening tool for identification of ALK rearrangement in non-small-cell lung carcinoma and is antibody dependent. J. Thorac. Oncol..

[52] Hofman P., Ilie M., Hofman V., Roux S., Valent A., Bernheim A., Alifano M., Leroy-Ladurie F., Vaylet F., Rouquette I. (2012). Immunohistochemistry to identify EGFR mutations or ALK rearrangements in patients with lung adenocarcinoma. Ann. Oncol..

[53] Hutarew G., Hauser-Kronberger C., Strasser F., Llenos I.C., Dietze O. (2014). Immunohistochemistry as a screening tool for ALK rearrangement in NSCLC: Evaluation of five different ALK antibody clones and ALK FISH. Histopathology.

[54] Ibrahim M., Parry S., Wilkinson D., Bilbe N., Allen D., Forrest S., Maxwell P., O’Grady A., Starczynski J., Tanier P. (2016). ALK immunohistochemistry in NSCLC: Discordant staining can impact patient treatment regimen. J. Thorac. Oncol..

[55] Gruber K., Kohlhäufl M., Friedel G., Ott G., Kalla C. (2015). A novel, highly sensitive ALK antibody 1A4 facilitates effective screening for ALK rearrangements in lung adenocarcinomas by standard immunohistochemistry. J. Thorac. Oncol..

[56] Wang Q., Zhao L., Yang X., Wei S., Zeng Y., Mao C., Lin L., Fu P., Lyu L., Li Z. (2016). Antibody 1A4 with routine immunohistochemistry demonstrates high sensitivity for ALK rearrangement screening of Chinese lung adenocarcinoma patients: A single-center large-scale study. Lung Cancer.

[57] Betz B.L., Dixon C.A., Weigelin H.C., Knoepp S.M., Roh M.H. (2013). The use of stained cytologic direct smears for ALK gene rearrangement analysis of lung adenocarcinoma. Cancer Cytopathol..

[58] Proietti A., Alì G., Pelliccioni S., Lupi C., Sensi E., Boldrini L., Servadio A., Chella A., Ribechini A., Cappuzzo F. (2014). Anaplastic lymphoma kinase gene rearrangements in cytological samples of non-small cell lung cancer: Comparison with histological assessment. Cancer Cytopathol..

[59] Zhong J., Li X., Bai H., Zhao J., Wang Z., Duan J., An T., Wu M., Wang Y., Wang S. (2016). Malignant pleural effusion cell blocks are substitutes for tissue in EML4-ALK rearrangement detection in patients with advanced non-small-cell lung cancer. Cytopathology.

[60] Zito Marino F., Rossi G., Brunelli M., Malzone M.G., Liguori G., Bogina G., Morabito A., Rocco G., Franco R., Botti G. (2017). Diagnosis of anaplastic lymphoma kinase rearrangement in cytological samples through a fluorescence in situ hybridization-based assay: Cytological smears versus cell blocks. Cancer.

[61] Long-Mira E., Washetine K., Hofman P. (2016). Sense and nonsense in the process of accreditation of a pathology laboratory. Virchows Arch..

[62] Minca E.C., Portier B.P., Wang Z., Lanigan C., Farver C.F., Feng Y., Ma P.C., Arrossi V.A., Pennell N.A., Tubbs R.R. (2013). ALK status testing in non-small cell lung carcinoma: Correlation between ultrasensitive IHC and FISH. J. Mol. Diagn..

[63] Minca E.C., Lanigan C.P., Reynolds J.P., Wang Z., Ma P.C., Cicenia J., Almeida F.A., Pennell N.A., Tubbs R.R. (2014). ALK status testing in non-small-cell lung carcinoma by FISH on thin prep slides with cytology material. J. Thorac. Oncol..

[64] Martin V., Bernasconi B., Merlo E., Balzarini P., Vermi W., Riva A., Chiaravalli A.M., Frattini M., Sahnane N., Facchetti F. (2015). ALK testing in lung adenocarcinoma: Technical aspects to improve FISH evaluation in daily practice. J. Thorac. Oncol..

[65] Von Laffert M., Stenzinger A., Hummel M., Weichert W., Lenze D., Warth A., Penzel R., Herbst H., Kellner U., Jurmeister P. (2015). ALK-FISH borderline cases in non-small cell lung cancer: Implications for diagnostics and clinical decision making. Lung Cancer.

[66] Gao X., Sholl L.M., Nishino M., Heng J.C., Jänne P.A., Oxnard G.R. (2015). Clinical implications of variant ALK FISH rearrangement patterns. J. Thorac. Oncol..

[67] Selinger C., Cooper W., Lum T., McNeil C., Morey A., Waring P., Amanuel B., Millward M., Peverall J., van Vliet C. (2015). Equivocal ALK fluorescence in situ hybridization (FISH) cases may benefit from ancillary ALK FISH probe testing. Histopathology.

[68] Li T., Maus M.K., Desai S.J., Beckett L.A., Stephens C., Huang E., Hsiang J., Zeger G., Danenberg K.D., Astrow S.H. (2014). Large-scale screening and molecular characterization of EML4-ALK fusion variants in archival non-small-cell lung cancer tumor specimens using quantitative reverse transcription polymerase chain reaction assays. J. Thorac. Oncol..

[69] Zhang X., Zhou J.G., Wu H.L., Ma H., Jiang Z.X. (2017). Diagnostic accuracy of PCR for detecting ALK gene rearrangement in NSCLC patients: A systematic review and meta-analysis. Oncotarget.

[70] Karachaliou N., Rosell R. (2013). Optimal detection of ALK rearranged lung adenocarcinomas. J. Thorac. Oncol..

[71] Inamura K., Takeuchi K., Togashi Y., Nomura K., Ninomiya H., Okui M., Satoh Y., Okumura S., Nakagawa K., Soda M. (2008). EML4-ALK fusion is linked to histological characteristics in a subset of lung cancers. J. Thorac. Oncol..

[72] Hiley C.T., Le Quesne J., Santis G., Sharpe R., de Castro D.G., Middleton G., Swanton C. (2016). Challenges in molecular testing in non-small-cell lung cancer patients with advanced disease. Lancet.

[73] Rozenblum A.B., Ilouze M., Dudnik E., Dvir A., Soussan-Gutman L., Geva S., Peled N. (2017). Clinical impact of hybrid capture-based next-generation sequencing on changes in treatment decisions in lung cancer. J. Thorac. Oncol..

[74] Chen H., Luthra R., Goswami R.S., Singh R.R., Roy-Chowdhuri S. (2015). Analysis of pre-analytic factors affecting the success of clinical next-generation sequencing of solid organ malignancies. Cancers.

[75] Devarakonda S., Masood A., Govindan R. (2017). Next-generation sequencing of lung cancers: Lessons learned and future directions. Hematol. Oncol. Clin. North. Am..

[76] Hagemann I.S., Devarakonda S., Lockwood C.M., Spencer D.H., Guebert K., Bredemeyer A.J., Al-Kateb H., Nguyen T.T., Duncavage E.J., Cottrell C.E. (2015). Clinical next-generation sequencing in patients with non-small cell lung cancer. Cancer.

[77] Luthra R., Chen H., Roy-Chowdhuri S., Singh R.R. (2015). Next-generation sequencing in clinical molecular diagnostics of cancer: Advantages and challenges. Cancers.

[78] Dagogo-Jack I., Shaw A.T. (2016). Screening for ALK rearrangements in lung cancer: Time for a new generation of diagnostics?. Oncologist.

[79] Goswami R.S., Luthra R., Singh R.R., Patel K.P., Routbort M.J., Aldape K.D., Yao H., Dang H.D., Barkoh B.A., Manekia J. (2016). Identification of factors affecting the success of next-generation sequencing testing in solid tumors. Am. J.Clin. Pathol..

[80] Dacic S., Villaruz L.C., Abberbock S., Mahaffey A., Incharoen P., Nikiforova M.N. (2016). ALK FISH patterns and the detection of ALK fusions by next generation sequencing in lung adenocarcinoma. Oncotarget.

[81] Koitzsch U., Heydt C., Attig H., Immerschitt I., Merkelbach-Bruse S., Fammartino A., Büttner R.H., Kong Y., Odenthal M. (2017). Use of the GeneReader NGS System in a clinical pathology laboratory: A comparative study. J. Clin. Pathol..

[82] Lindeman N.I., Cagle P.T., Beasley M.B., Chitale D.A., Dacic S., Giaccone G., Jenkins R.B., Kwiatkowski D.J., Saldivar J.S., Squire J. (2013). Molecular testing guideline for selection of lung cancer patients for EGFR and ALK tyrosine kinase inhibitors: Guideline from the college of american pathologists, international association for the study of lung cancer, and association for molecular pathology. J. Thorac. Oncol..

[83] Evangelista A.F., Zanon M.F., Carloni A.C., de Paula F.E., Morini M.A., Ferreira-Neto M., Soares I.C., Miziara J.E., de Marchi P., Scapulatempo-Neto C. (2017). Detection of ALK fusion transcripts in FFPE lung cancer samples by NanoString Technology. BMC Pulm. Med..

[84] Geiss G.K., Bumgarner R.E., Birditt B., Dahl T., Dowidar N., Dunaway D.L., Fell H.P., Ferree S., George R.D., Grogan T. (2008). Direct multiplexed measurement of gene expression with color-coded probe pairs. Nat. Biotechnol..

[85] Lira M.E., Choi Y.L., Lim S.M., Deng S., Huang D., Ozeck M., Han J., Jeong J.Y., Shim H.S., Cho B.C. (2014). A single-tube multiplexed assay for detecting ALK, ROS1, and RET fusions in lung cancer. J. Mol. Diagn..

[86] Nakajima N., Yoshizawa A., Kondo K., Rokutan-Kurata M., Hirata M., Furuhata A., Sumiyoshi S., Sonobe M., Menju T., Momose M. (2017). Evaluating the effectiveness of RNA in situ hybridization for detecting lung adenocarcinoma with anaplastic lymphoma kinase rearrangement. Histopathology.

[87] Schildhaus H.U., Deml K.F., Schmitz K., Meiboom M., Binot E., Hauke S., Merkelbach-Bruse S., Büttner R. (2013). Chromogenic in situ hybridization is a reliable assay for detection of ALK rearrangements in adenocarcinomas of the lung. Mod. Pathol..

[88] Wang F., Flanagan J., Su N., Wang L.C., Bui S., Nielson A., Wu X., Vo H.T., Ma X.J., Luo Y. (2012). RNAscope: A novel in situ RNA analysis platform for formalin-fixed, paraffin-embedded tissues. J. Mol. Diagn..

[89] Roussel H., de Guillebon E., Biard L., Mandavit M., Gibault L., Fabre E., Antoine M., Hofman P., Beau-Faller M., Blons H. (2017). Composite biomarkers defined by multiparametric immunofluorescence analysis identify ALK-positive adenocarcinoma as a potential target for immunotherapy. Oncoimmunology.

[90] Kim H., Yoo S.B., Choe J.Y., Paik J.H., Xu X., Nitta H., Zhang W., Grogan T.M., Lee C.T., Jheon S. (2011). Detection of ALK gene rearrangement in non-small cell lung cancer: A comparison of fluorescence in situ hybridization and chromogenic in situ hybridization with correlation of ALK protein expression. J. Thorac. Oncol..

[91] Martinez P., Hernández-Losa J., Montero M.Á., Cedrés S., Castellví J., Martinez-Marti A., Tallada N., Murtra-Garrell N., Navarro-Mendivill A., Rodriguez-Freixinos V. (2013). Fluorescence in situ hybridization and immunohistochemistry as diagnostic methods for ALK positive non-small cell lung cancer patients. PLoS ONE.

[92] McLeer-Florin A., Moro-Sibilot D., Melis A., Salameire D., Lefebvre C., Ceccaldi F., de Fraipont F., Brambilla E., Lantuejoul S. (2012). Dual IHC and FISH testing for ALK gene rearrangement in lung adenocarcinomas in a routine practice: A French study. J. Thorac. Oncol..

[93] Mino-Kenudson M., Chirieac L.R., Law K., Hornick J.L., Lindeman N., Mark E.J., Cohen D.W., Johnson B.E., Jänne P.A., Iafrate A.J. (2010). A novel, highly sensitive antibody allows for the routine detection of ALK-rearranged lung adenocarcinomas by standard immunohistochemistry. Clin. Cancer Res..

[94] Ying J., Guo L., Qiu T., Shan L., Ling Y., Liu X., Lu N. (2013). Diagnostic value of a novel fully automated immunochemistry assay for detection of ALK rearrangement in primary lung adenocarcinoma. Ann. Oncol..

[95] Jiang L., Yang H., He P., Liang W., Zhang J., Li J., Liu Y., He J. (2016). Improving selection criteria for ALK inhibitor therapy in non-small cell lung cancer: A pooled-data analysis on diagnostic operating characteristics of immunohistochemistry. Am. J. Surg. Pathol..

[96] Alì G., Proietti A., Pelliccioni S., Niccoli C., Lupi C., Sensi E., Giannini R., Borrelli N., Menghi M., Chella A. (2014). ALK rearrangement in a large series of consecutive non-small cell lung cancers: Comparison between a new immunohistochemical approach and fluorescence in situ hybridization for the screening of patients eligible for crizotinib treatment. Arch. Pathol. Lab. Med..

[97] Cabillic F., Gros A., Dugay F., Begueret H., Mesturoux L., Chiforeanu D.C., Dufrenot L., Jauffret V., Dachary D., Corre R. (2014). Parallel FISH and immunohistochemical studies of ALK status in 3244 non-small-cell lung cancers reveal major discordances. J. Thorac. Oncol..

[98] Heuckmann J.M., Pauwels P., Thunnissen E. (2017). Comprehensive hybrid capture-based next-generation sequencing identifies a double ALK gene fusion in a patient previously identified to be false-negative by FISH. J. Thorac. Oncol..

[99] Lantuejoul S., Rouquette I., Blons H., Le Stang N., Ilie M., Begueret H., Grégoire V., Hofman P., Gros A., Garcia S. (2015). French multicentric validation of ALK rearrangement diagnostic in 547 lung adenocarcinomas. Eur. Respir. J..

[100] Li W., Zhang J., Guo L., Chuai S., Shan L., Ying J. (2017). Combinational analysis of FISH and immunohistochemistry reveals rare genomic events in ALK fusion patterns in NSCLC that responds to crizotinib treatment. J. Thorac. Oncol..

[101] Kim H., Xu X., Yoo S.B., Sun P.L., Jin Y., Paik J.H., Choe G., Jheon S., Lee C.T., Chung J.H. (2013). Discordance between anaplastic lymphoma kinase status in primary non-small-cell lung cancers and their corresponding metastases. Histopathology.

[102] Trejo Bittar H.E., Luvison A., Miller C., Dacic S. (2017). A comparison of ALK gene rearrangement and ALK protein expression in primary lung carcinoma and matched metastasis. Histopathology.

[103] Wiesner T., Lee W., Obenauf A.C., Ran L., Murali R., Zhang Q.F., Wong E.W., Hu W., Scott S.N., Shah R.H. (2015). Alternative transcription initiation leads to expression of a novel ALK isoform in cancer. Nature.

[104] Marchetti A., Di Lorito A., Pace M.V., Iezzi M., Felicioni L., D’Antuono T., Filice G., Guetti L., Mucilli F., Buttitta F. (2016). ALK protein analysis by IHC staining after recent regulatory changes: A comparison of two widely used approaches, revision of the literature, and a new testing algorithm. J. Thorac. Oncol..

[105] Rosoux A., Pauwels P., Duplaquet F., D’Haene N., Weynand B., Delos M., Menon R., Heukamp L.C., Thunnissen E., Ocak S. (2016). Effectiveness of crizotinib in a patient with ALK IHC-positive/FISH-negative metastatic lung adenocarcinoma. Lung Cancer.

[106] Ilie M., Hofman P. (2015). Reply to the letter to the editor ’ALK FISH rearranged and amplified tumor with negative immunohistochemistry: A rare and challenging case concerning ALK status screening in lung cancer’ by Uguen et al. Ann. Oncol..

[107] Uguen A., Talagas M., Andrieu-Key S., Costa S., Quintin-Roué I., De Braekeleer M., Marcorelles P. (2015). ALK FISH rearranged and amplified tumor with negative immunohistochemistry: A rare and challenging case concerning ALK status screening in lung cancer. Ann. Oncol..

[108] Roth A., Streubel A., Grah C., Stephan-Falkenau S., Mairinger T., Wagner F. (2014). A rare case of an EML4-ALK-rearranged lung adenocarcinoma missed by in situ-hybridization but detected by RT-PCR. J. Clin. Pathol..

[109] Pekar-Zlotin M., Hirsch F.R., Soussan-Gutman L., Ilouze M., Dvir A., Boyle T., Wynes M., Miller V.A., Lipson D., Palmer G.A. (2015). Fluorescence in situ hybridization, immunohistochemistry, and next-generation sequencing for detection of EML4-ALK rearrangement in lung cancer. Oncologist.

[110] Peled N., Palmer G., Hirsch F.R., Wynes M.W., Ilouze M., Varella-Garcia M., Soussan-Gutman L., Otto G.A., Stephens P.J., Ross J.S. (2012). Next-generation sequencing identifies and immunohistochemistry confirms a novel crizotinib-sensitive ALK rearrangement in a patient with metastatic non-small-cell lung cancer. J. Thorac. Oncol..

[111] Teixidó C., Karachaliou N., Peg V., Gimenez-Capitan A., Rosell R. (2014). Concordance of IHC, FISH and RT-PCR for EML4-ALK rearrangements. Transl. Lung Cancer Res..

[112] Reguart N., Teixidó C., Giménez-Capitán A., Paré L., Galván P., Viteri S., Rodríguez S., Peg V., Aldeguer E., Viñolas N. (2017). Identification of ALK, ROS1, and RET fusions by a multiplexed mRNA-based assay in formalin-fixed, paraffin-embedded samples from advanced non-small-cell lung cancer patients. Clin. Chem..

[113] Rogers T.M., Arnau G.M., Ryland G.L., Huang S., Lira M.E., Emmanuel Y., Perez O.D., Irwin D., Fellowes A.P., Wong S.Q. (2017). Multiplexed transcriptome analysis to detect ALK, ROS1 and RET rearrangements in lung cancer. Sci. Rep..

[114] Sunami K., Furuta K., Tsuta K., Sasada S., Izumo T., Nakaoku T., Shimada Y., Saito M., Nokihara H., Watanabe S. (2016). Multiplex diagnosis of oncogenic fusion and MET exon skipping by molecular counting using formalin-fixed paraffin embedded lung adenocarcinoma tissues. J. Thorac. Oncol..

[115] Wynes M.W., Sholl L.M., Dietel M., Schuuring E., Tsao M.S., Yatabe Y., Tubbs R.R., Hirsch F.R. (2014). An international interpretation study using the ALK IHC antibody D5F3 and a sensitive detection KIT demonstrates high concordance between ALK IHC and ALK FISH and between evaluators. J. Thorac. Oncol..

[116] Shan L., Lian F., Guo L., Yang X., Ying J., Lin D. (2014). Combination of conventional immunohistochemistry and qRT-PCR to detect ALK rearrangement. Diagn. Pathol..

[117] Kerr K.M., López-Ríos F. (2016). Precision medicine in NSCLC and pathology: How does ALK fit in the pathway?. Ann. Oncol..

[118] Aisner D.L., Rumery M.D., Merrick D.T., Kondo K.L., Nijmeh H., Linderman D.J., Doebele R.C., Thomas N., Chesnut P.C., Varella-Garcia M. (2016). Do more with less: Tips and techniques for maximizing small biopsy and cytology specimens for molecular and ancillary testing: The university of colorado experience. Arch. Pathol. Lab. Med..

[119] Yatabe Y. (2015). ALK FISH and IHC: You cannot have one without the other. J. Thorac. Oncol..

[120] Houang M., Toon C.W., Clarkson A., Sioson L., Watson N., Farzin M., Selinger C.I., Chou A., Morey A.L., Cooper W.A. (2014). Reflex ALK immunohistochemistry is feasible and highly specific for ALK gene rearrangements in lung cancer. Pathology.

[121] Marchetti A., Pace M.V., Di Lorito A., Canarecci S., Felicioni L., D’Antuono T., Liberatore M., Filice G., Guetti L., Mucilli F. (2016). Validation of a new algorithm for a quick and easy RT-PCR-based ALK test in a large series of lung adenocarcinomas: Comparison with FISH, immunohistochemistry and next generation sequencing assays. Lung Cancer.

[122] Thorne-Nuzzo T., Williams C., Catallini A., Clements J., Singh S., Amberson J., Dickinson K., Gatalica Z., Ho S.N., Loftin I. (2017). A sensitive ALK immunohistochemistry companion diagnostic test identifies patients eligible for treatment with crizotinib. J. Thorac. Oncol..

[123] Van der Wekken A.J., Pelgrim R., ’t Hart N., Werner N., Mastik M.F., Hendriks L., Van der Heijden E.H.F.M., Looijen-Salamon M., De Langen A.J., Staal-van den Brekel J. (2017). Dichotomous ALK-IHC is a better predictor for ALK inhibition outcome than traditional ALK-FISH in advanced non-small cell lung cancer. Clin. Cancer Res..

[124] Von Laffert M., Schirmacher P., Warth A., Weichert W., Büttner R., Huber R.M., Wolf J., Griesinger F., Dietel M., Grohé C. (2017). ALK-Testing in non-small cell lung cancer (NSCLC): Immunohistochemistry (IHC) and/or fluorescence in situ Hybridisation (FISH)?: Statement of the Germany Society for Pathology (DGP) and the Working Group Thoracic Oncology (AIO) of the German Cancer Society e.V. (Stellungnahme der Deutschen Gesellschaft für Pathologie und der AG Thorakale Onkologie der Arbeitsgemeinschaft Onkologie/Deutsche Krebsgesellschaft e.V.). Lung Cancer.

[125] Vinciguerra G.L.R., Scarpino S., Pini B., Cippitelli C., Fochetti F., Ruco L. (2017). Optimized immunohistochemistry using the D5F3 antibody provides a reliable test for identification of ALK-positive lung adenocarcinomas. Virchows Arch..

[126] Jørgensen J.T., Hersom M. (2016). Companion diagnostics-a tool to improve pharmacotherapy. Ann. Transl. Med..

[127] Jürgens J., Engel-Riedel W., Stoelben E., Schildgen V., Schildgen O., Brockmann M. (2016). The (con-) fusion in ALK diagnostics: When food and drug administration-approved algorithms fail. J. Clin. Oncol..

[128] Thunnissen E., Allen T.C., Adam J., Aisner D.L., Beasley M.B., Borczuk A.C., Cagle P.T., Capelozzi V.L., Cooper W., Hariri L.P. (2017). Immunohistochemistry of pulmonary biomarkers: A perspective from members of the Pulmonary Pathology Society. Arch. Pathol. Lab. Med..

[129] Li Y., Zhang R., Peng R., Ding J., Han Y., Wang G., Zhang K., Lin G., Li J. (2016). Reliability assurance of detection of EML4-ALK rearrangement in non-small cell lung cancer: The results of proficiency testing in China. J. Thorac. Oncol..

[130] Marchetti A., Barberis M., Papotti M., Rossi G., Franco R., Malatesta S., Buttitta F., Ardizzoni A., Crinò L., Gridelli C. (2014). ALK rearrangement testing by FISH analysis in non-small-cell lung cancer patients: Results of the first italian external quality assurance scheme. J. Thorac. Oncol..

[131] Nielsen S. (2015). External quality assessment for immunohistochemistry: Experiences from NordiQC. Biotech. Histochem..

[132] Tembuyser L., Tack V., Zwaenepoel K., Pauwels P., Miller K., Bubendorf L., Kerr K., Schuuring E., Thunnissen E., Dequeker E.M. (2014). The relevance of external quality assessment for molecular testing for ALK positive non-small cell lung cancer: Results from two pilot rounds show room for optimization. PLoS ONE.

